# Real-world data on adjuvant capecitabine after standard neoadjuvant chemotherapy for triple negative breast cancer

**DOI:** 10.61622/rbgo/2024rbgo29

**Published:** 2024-07-26

**Authors:** Maria Fernanda Imperio Pereira, Isabela Panzeri Carlotti Buzatto, Hélio Humberto Angotti Carrara, Fabiana de Oliveira Buono, Jurandyr Moreira de Andrade, Leonardo Fleury Orlandini, Daniel Guimarães Tiezzi

**Affiliations:** 1 Department of Gynecology and Obstetrics Ribeirão Preto Medical School Universidade de São Paulo Ribeirão Preto SP Brazil Department of Gynecology and Obstetrics, Ribeirão Preto Medical School, Universidade de São Paulo, Ribeirão Preto, SP, Brazil.; 2 Advanced Research Center in Medicine União das Faculdades dos Grandes Lagos São José do Rio Preto SP Brazil Advanced Research Center in Medicine, União das Faculdades dos Grandes Lagos, São José do Rio Preto, SP, Brazil.

**Keywords:** Breast neoplasms, Neoadjuvant therapy, Triple-negative breast neoplasms, Capecitabine/therapeutic use

## Abstract

**Objective:**

Neoadjuvant chemotherapy (NACT) has become the standard of care for patients with triple-negative breast cancer (TNBC) with tumors > 1 cm or positive axillary nodes. Pathologic complete response (pCR) has been used as an endpoint to select patients for treatment scaling. This study aimed to examine the benefit of adding adjuvant capecitabine for TNBC patients who did not achieve pCR after standard NACT in a real-world scenario.

**Methods:**

This retrospective cohort study included all patients with TNBC who underwent NACT between 2010 and 2020. Clinicopathological data were obtained from the patient records. Univariate and multivariate analyses were conducted at the 5 years follow-up period.

**Results:**

We included 153 patients, more than half of whom had stage III (58.2%) and high-grade tumors (60.8%). The overall pCR rate was 34.6%, and 41% of the patients with residual disease received adjuvant capecitabine. Disease-specific survival (DSS) among the patients who achieved pCR was significantly higher (p<0.0001). Residual disease after NACT was associated with detrimental effects on DSS. In this cohort, we did not observe any survival benefit of adding adjuvant capecitabine for patients with TNBC subjected to NACT who did not achieve pCR (p=0.52).

**Conclusion:**

Our study failed to demonstrate a survival benefit of extended capecitabine therapy in patients with TNBC with residual disease after NACT. More studies are warranted to better understand the indication of systemic treatment escalation in this scenario.

## Introduction

Breast cancer is a heterogeneous disease with different etiologies, clinicopathological characteristics, and responses to treatment.^(1)^Breast cancer is currently divided into five subtypes, derived from the Perou et al. classification (2000).^(2)^Based on immunohistochemistry, tumors expressing the estrogen receptor (ER) and/or progesterone receptor (PR) are luminal-like breast cancers. Tumors expressing human epidermal growth factor receptor 2 (HER2) are HER2 enriched (whether or not luminal), and tumors that lack the expression of hormone receptors and HER2 enrichment are triple-negative breast cancer (TNBC).^(3)^

TNBC represents 15-20% of all diagnosed breast cancers and is known to be a more aggressive disease with poorer prognosis and higher mortality than the other subtypes.^(4,5)^TNBC is the most chemotherapy sensitive among subtypes, and anthracycline and taxane based regimens remain the standard of care. Despite this, there is no standard therapy for refractory or relapsed disease, although new treatment options have recently emerged.^(4)^

Neoadjuvant chemotherapy (NACT) has become the standard of care for TNBC tumors greater than 1 cm (T1b), since this allows *in vivo* evaluation of the effectiveness of systemic therapy. A pathological complete response (pCR) to NACT has been associated with increased disease-free survival (DFS) and could be considered a suitable marker of survival.^(6)^ Notwithstanding this, women who do not achieve pCR are candidates for personalized adjuvant therapy.^(4,7)^

Capecitabine is an oral prodrug of fluorouracil, which is mainly prescribed for the treatment of advanced breast cancer.^(8)^A recent meta-analysis suggested that the addition of capecitabine in combination or in sequence with standard chemotherapy is associated with a modest benefit in DFS and overall survival (OS).^(9)^However, there has been a worldwide increase in the use of capecitabine in the adjuvant setting for women with residual disease after standard NACT. A single prospective study named the CREATE-X trial showed the benefit of adding capecitabine as an extended therapy in TNBC patients with no pCR after NACT.^(10)^The GEICAM/2003-11_CIBOMA/2004-01 trial also evaluated clinical benefit of adjuvant capecitabine for TNBC patients. However, the authors did not find a significant benefit related to this drug. In fact, they have shown an improvement in DFS in a subgroup of TNBC characterized as non-basal tumors based on the lack of staining for epidermal growth factor receptor and cytokeratin 5/6 expression in immunohistochemistry analysis.^(11)^ The benefit was observed only in the CREATE-X trial, and the fact that they only included exclusively Asian women is a current source of controversy.

The aim of this study was to examine, in a population often diagnosed with advanced stages, the benefit of the addition of adjuvant capecitabine in patients with TNBC who did not achieve pCR after standard NACT.

## Methods

We performed a retrospective cohort study which included all patients diagnosed with TNBC who underwent NACT at the Clinics Hospital of Ribeirão Preto School of Medicine, University of São Paulo (USP), between 2010 and 2020. Clinical and pathological data were obtained from patients’ files (including paper charts and electronic medical records).

The criteria for TNBC classification were based on immunohistochemical data in the pathology reports of core needle biopsy performed before breast cancer treatment. We considered estrogen receptor-negative and progesterone receptor-negative tumors to have less than 1% protein expression.^(12)^HER2 status was established in accordance with the pathology report and protocols followed at the time of diagnosis, as recorded in the clinical chart.^(13)^For staging, we used the American Joint Committee on Cancer Staging Manual, 7th edition.^(14)^

A pCR was considered if there was no residual invasive carcinoma in the breast or axilla after NACT. Patients with residual invasive carcinoma were characterized as having residual disease (RD). Patients in the RD group were divided according to the use of extended adjuvant chemotherapy into the capecitabine or no adjuvant (NoAdj) groups. Toxicity and reports of delayed or canceled treatment with capecitabine were retrieved from medical files.

The exclusion criteria were metastatic disease, previous malignant breast disease, simultaneous non-triple-negative tumor, changes in the IHC classification after NACT interfering with systemic therapy, and death during NACT. A total of 158 patients were retrieved. Applying the exclusion criteria, one tumor was classified as HER2+ after NACT, two patients had synchronic bilateral non-triple negative tumors and two patients have died during the neoadjuvant treatment; all cases were excluded.

To evaluate the differences between the groups, we used the chi-square test for categorical variables and the Mann-Whitney or Student t tests for continuous variables based on the variable distribution. Normal distribution was inferred based on the histograms and tested by the Shapiro-Wilk test. We used a Cox multivariable regression model to analyze the variables considered significant in the univariate analyses. Kaplan-Meier curves were used to estimate survival, and the differences between the groups were tested using the log-rank test. The median follow up time was 5.4 years (IQR= 3.7 years). As capecitabine therapy started in 2017, the median follow up time in the Capecitabine group was 3.9 years (IQR= 1.8). Survival analyses were applied for the 5 years-follow-up in the whole dataset and were set to 4 years to analyze the impact of capecitabine therapy. The level of significance was set at 0.05, and all analyses were conducted using R software version 4.1.2 (R Core Team 2021, Vienna, Austria).

The study was approved by the Ethical Committee of the Institution 6141120, and the requirement for informed consent was waived (approval number 38438620.3.0000.5440).

## Results

The cohort included 153 retrospectively selected patients who underwent NACT for TNBC. Their mean age at diagnosis was 48.9±12 years (range 25-74 years). More than half of the women were diagnosed with stage III (58.2%) cancer, and 60.8% of the patients had grade 3 tumors. Regarding the systemic treatment schemes, most patients received anthracycline- and taxane-based chemotherapy (92.8%). We observed a pCR rate of 34.6%, and 41% of patients with residual disease received capecitabine as extended adjuvant treatment. Table 1 summarizes the patient characteristics.

**Table 1:** Patients’ characteristics of triple-negative breast cancer (TNBC) patients subjected to neoadjuvant chemotherapy (NACT)

Variables	n(%)
Age in years (mean ± sd)	48.9 ± 12
Cancer Stage (n)	
IIa	23(15)
IIb	41(26.8)
IIIa	39(25.5)
IIIb	45(29.4)
IIIc	5(3.3)
Clinical T size in mm (median ± mad)	52 ± 26.7
Histology	
Invasive ductal carcinoma	139(90.8)
Other	14(9.2)
Grade	
1	5(3.3)
2	55(35.9)
3	93(60.8)
NACT scheme	
Taxane-anthracycline based	142(92.8)
Anthracycline based	8(5.2)
Other	3(2)
Clinical response to NACT	
Complete	54(35.3)
Partial	56(36.6)
Stable	37(24.1)
Progression	6(3.9)
Pathological response to NACT	
Breast	
Complete	59(38.6)
Residual	94(61.4)
Breast + Axilla	
Complete	53(34.6)
Residual	100(65.4)
Axillary status after NACT	
Axilla	
Negative	105(68.6)
Positive	48(31.4)
Extended adjuvant chemotherapy	
Yes	41(26.8)
No	112(73.2)

mad - Median absolute deviation

Patients with smaller tumors and fewer metastatic lymph nodes at diagnosis more frequently achieved pCR, with 51% of T1/2 patients achieving pCR, in contrast to the 25% pCR ratio in T3/4 (p=0.006). Axillary involvement was also associated with residual disease (p=0.04). There was a tendency for younger women and tumors with higher Ki67 levels at initial biopsy to achieve pCR (p=0.09 and p=0.06), respectively). Table 2 summarizes the predictive factors for the response to NACT.

**Table 2:** Predictive factors of response to neoadjuvant chemotherapy (NACT) in 153 triple negative breast cancer (TNBC) patients

Variables	pCR n(%)	no- pCR n(%)	p-value
Age (median ± mad)	45.1 ± 11.7	51 ± 12.5	0.09
Age group			
< 40 years old	18(45)	22(55)	
≥ 40 years old	35(31.2)	77(68.8)	0.1
cT (median ± mad)	40 ± 22.2	59 ± 20.8	0.002
T			
1/2	29(50)	29(50)	
3	12(22.6)	41(77.4)	
4	12(28.4)	30(71.4)	0.006
0	26(47.3)	29(52.7)	
1	18(30)	42(70)	
2 or 3	9(23.7)	29(76.3)	0.04
Grade			
1 or 2	25(41.7)	35(58.3)	
3	28(30.1)	65(79.9)	0.2
KI67 (median; IQR)	70(30)	60(50)	0.06

pCR - Pathological complete response; mad - Median absolute deviation; IQR - interquartile range

**Figure 1:**
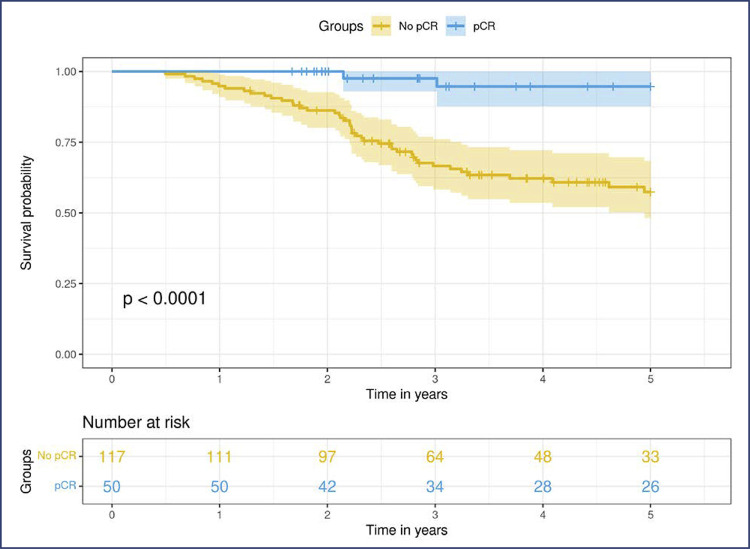
Five-year follow up for disease specific survival of patients who achieved or not pathological complete response (pCR)

We analyzed the effect of pCR on disease-specific survival (DSS) at the 5 years follow-up. Patients who achieved pCR had a higher probability of survival. However, the presence of residual disease after NACT is associated with a detrimental effect on DSS. The difference in survival probability was significant (p<0.0001), with a hazard ratio (HR) of 0.078 (CI95%, 0.02 to 0.32) in favor of patients with pCR. Figure 1 shows DSS at the 5-year follow-up.

In the univariate analysis, we observed that the clinical T size (HR= 1.027; 95%CI 1.018-1.037), the residual T size (HR= 1.018; 95% CI 1.013-1.023), axillary involvement (for ypN1 the HR= 3.4 (95%CI 1.4-7.8), and extensive post-NACT axillary involvement (ypN2/3) (HR= 9.3 ;95%CI 4.6-18.8) were significantly associated with a decrease in DSS.

### Extended adjuvant chemotherapy

A total of 100 patients with residual disease (RD) were divided into two groups: 59 patients did not receive extended adjuvant chemotherapy (NoAdj group) and 41 patients received 6 or 8 cycles of capecitabine (capecitabine group) after breast surgery. We investigated the adverse effects of capecitabine and their impact during treatment. A total of 28 patients (68.3%) experienced adverse effects. In eight patients, the capecitabine dose had to be reduced, and in five patients, there was a delay in the course of drug administration due to side effects. Eleven patients (27%) did not complete the full capecitabine regimen, and the median number of cycles was 6 (range, 2–8). The most frequent side effect was hand-foot syndrome (53%), followed by diarrhea and neutropenia (9.7% each), and 14.6% of the patients experienced a toxicity grade of 3 or 4. We analyzed the effect of adding extended adjuvant chemotherapy with capecitabine on DSS in the group of patients with no pCR. Table 3 summarizes patient characteristics. In addition to the higher Ki67 expression in tumors from the NoAdj group (p=0.04), we did not find any significant differences in the clinical and pathological features between the groups.

**Table 3:** Characteristics of patients that did not achieve complete response after neoadjuvant chemotherapy

Characteristics of patients	Capecitabine	NoAdj	p-value
Age (mean ± sd)	49.8 ± 11.4	50 ± 11.7	0.9
cT (median ± mad)	53 ± 19.3	60 ± 16.1	0.6
T			
1 or 2	15(36.6)	14(23.7)	
3	17(41.5)	24(40.7)	
4	9(22)	21(35.6)	0.2
0	13(31.7)	16(27.1)	
1	19(46.3)	23(39)	
2 or 3	9(22)	20(33.9)	0.4
Grade			
1 or 2	14(34.1)	21(35.6)	
3	27(65.9)	38(64.4)	1
KI67 (median ± mad)	50 ± 37	70 ± 29.6	0.02
ypT (median ± mad)	16 ± 14.8	23 ± 27.1	0.3
ypN (%)			
0	23(56.1)	30(50.8)	
1	8(22)	15(25.4)	
2 or 3	8(22)	14(23.7)	0.4

mad - Median absolute deviation

**Figure 2:**
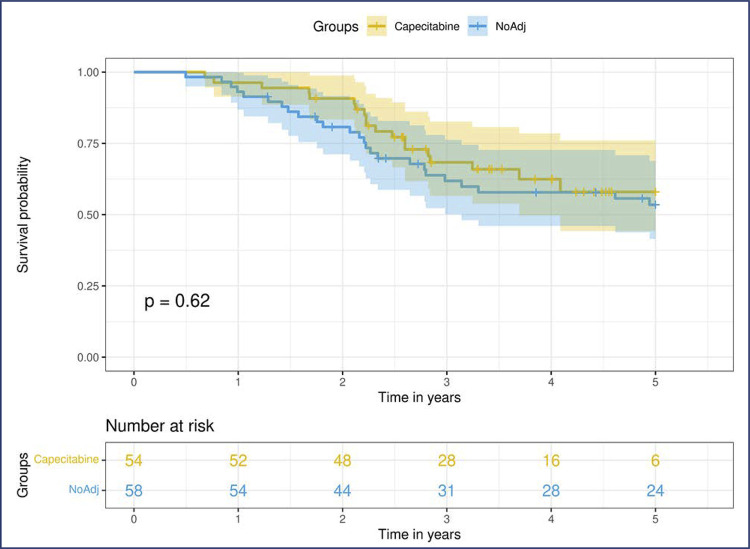
Five-year follow-up for disease specific survival of patients who received or did not receive adjuvant capecitabine

DSS in the Capecitabine and NoAdj groups was not significantly different (p=0.51). Figure 2 shows the Kaplan–Meier curves. We investigated the influence of the clinical and pathological features of DSS in patients without pCR. Clinical T size (HR= 1.02; 95%CI= 1.015-1.035), residual T size (HR= 1.015; 95%CI= 1.008-1.02), and the presence of residual axillary lymph nodes (HR= 4.8; 95%CI= 2.2-10.3 for ypN2/3) were significant prognostic factors in univariate analysis. We applied a multivariable model including the groups (Capecitabine and NoAdj) and their significant predictors in the univariate analysis. Table 4 summarizes the statistical analyses of the Cox model.

**Table 4:** Multivariable analysis of disease specific survival of patients who did not achieve complete response after neoadjuvant chemotherapy

Variables	HR	95%CI	p-value
Clinical T size (mm)	1.017	1.005-1.03	0.003
ypT size (mm)	1.01	1.001-1.02	0.02
ypN (reference= 0)			
1	2.6	1.06-6.6	0.03
2 or 3	4.6	2.1-10	0.0001
Groups (reference= noAdj)			
Capecitabine	0.9	0.4-1.8	0.7

## Discussion

Delivering NACT has recently become the standard therapeutic approach for early stage TNBC larger than 1 cm or with positive nodes.^(15)^In addition to the benefit of diminishing the primary tumor size, this rationale is based on the use of the pathological response to treatment as an endpoint to select patients for treatment escalation. In this retrospective study, our results showed that extended chemotherapy with adjuvant capecitabine was not associated with a statistically significant benefit in terms of overall and DSS in TNBC patients who underwent NACT with no pCR. To our knowledge, this is the first study to address this issue in the Brazilian population.

The rate of pCR after NACT is approximately 37% with chemotherapy regimens that do not contain carboplatin or pembrolizumab.^(16)^We observed an overall pCR rate of 34.6% in our study, which is in accordance with data from the literature.^(17)^ Achieving pCR improves prognoses to the point that their DSS and OS are similar to those of patients with luminal tumors who received chemotherapy.^(6)^ Indeed, we observed a statistically significant higher DSS among patients who achieved complete response after neoadjuvant chemotherapy than among those with residual disease in the breast and/or axilla. We also observed a decrease in DSS when extensive disease was present at diagnosis and/or bulky residual disease was identified in the breast or axilla after NACT. Therefore, patients with TNBC and residual disease after NACT, especially those with high-volume residual disease, deserve a different approach in the adjuvant setting than those that achieve a pCR.

Two recent studies addressed the escalation of treatment with adjuvant capecitabine. The phase III CREATE-X trial published in 2017 was a practice change. They randomized approximately 900 patients to capecitabine versus observation after NACT and no pCR (30% were TNBC). Subgroup analysis of the TNBC patients demonstrated a beneficial survival rate in the capecitabine-treated group.^(10)^ The results of this trial are therefore not easily reproducible worldwide. The multicenter phase III GEICAM/2003-11_CIBOMA/2004-01 trial, published in 2019, failed to demonstrate a statistically significant increase in DSS by adding extended adjuvant capecitabine in the treatment of patients with early stage TNBC. Only 20% of the included patients received NACT.^(11)^

Recently, an individual patient data meta-analysis, including more than 15,000 patients, showed that the addition of capecitabine to systemic treatment improved DSS and OS in TNBC.^(9)^However, the authors suggest that most of the meta-analysis effect was driven by CREATE-X trial, and the actual effect of adding capecitabine should be small; therefore, clinicians should individualize its prescription. Our real-world results showed that extended chemotherapy with capecitabine was not associated with a significant DSS benefit in this subset of patients. It is important to highlight that the CREATE-X trial was conducted exclusively with Asian women. This could explain the “too good to be true” findings of the study.^(18)^It is already known that Asian women have differences in pharmacogenomics and pharmacokinetics when metabolizing capecitabine compared to Caucasian women. This was reported in the Xeloda® package insert and has been previously published.^(19)^

Capecitabine is not free of adverse effects (AE). In the aforementioned trials, patients had grade 3 or 4 adverse effects in up to 18% of cases. Similar to our findings, the most common side effect was hand-foot syndrome, followed by neutropenia and diarrhea.^(10,11)^ In addition to the toxicity profile, the cost of the broad use of a medication with conflicting results in this scenario, mainly in middle- and low-income countries, should be considered.

Although pCR has been frequently used as a surrogate end point for neoadjuvant trials in early breast cancer settings, data on patient survival, compared to patients who received surgery upfront and adjuvant chemotherapy (AC), are still conflicting.^(20,21)^A recent meta-analysis including more than 36,442 TNBC patients suggested that the OS was higher in women who received AC than in those who received NACT. The detrimental effects on OS and DFS were specifically related to RD after NACT.^(22)^This observation may be explained by the selection bias of more aggressive tumors to NACT, or by the earlier tumor debulking by primary surgery diminishing the risk of tumor seeding and systemic metastasis in TNBC resistant to chemotherapy.^(23)^ The need to resolve this issue is extremely relevant to the definition of treatment in patients with operable TNBC.

A recent report demonstrated a clear trend in favor of NACT over AC for all early-stage breast cancer subtypes in Germany.^(24)^They showed that the proportion of patients receiving NACT increased from 24.6% in 2008 to 76.2% in 2017, the same year that the CREATE-X trial was published. We believe that the proportion of patients with early-stage breast cancer treated with NACT has been increasing ever since. Based on the assumption that the delay in tumor resection in patients with chemotherapy-resistant TNBC may be detrimental in terms of OS and DFS, delivering NACT for early stage TNBC would be harmful. Therefore, the estimation of the real benefit of extended chemotherapy in this scenario must be fully confirmed.

A limitation of our study is that it was an observational retrospective study with a risk of data collection bias. On the other hand, it was developed in a university hospital where patients are systematically followed up and all patients’ files are kept in an electronic health system. Although the study was based on convenience sampling and the number of events was not sufficient to make inferences for short-term follow-up (up to 2 years), the analyses at 4- and 5-years follow-up were robust enough for statistical inferences. Notwithstanding, this is a hypothesis-generating study in which we question the real benefit of scaling systemic treatment with possible toxicity, based on only one study in a group of only Asian women and with conflicting data in the literature.

## Conclusion

Our results confirmed that TNBC is an aggressive subtype of breast cancer and that pCR is an important surrogate endpoint. However, we failed to demonstrate a survival benefit of adding capecitabine in the adjuvant setting in women with TNBC and residual disease after NAC. Further prospective trials should focus on selecting patients who can benefit from this adjuvant strategy, particularly in countries with limited public health budgets.
